# Visualizing the Distribution of Synapses from Individual Neurons in the Mouse Brain

**DOI:** 10.1371/journal.pone.0011503

**Published:** 2010-07-09

**Authors:** Ling Li, Bosiljka Tasic, Kristina D. Micheva, Vsevolod M. Ivanov, Maria L. Spletter, Stephen J. Smith, Liqun Luo

**Affiliations:** 1 Howard Hughes Medical Institute, Department of Biology, Stanford University, Stanford, California, United States of America; 2 Department of Molecular and Cellular Physiology, Stanford University, Stanford, California, United States of America; 3 Lynbrook High School, San Jose, California, United States of America; Columbia University, United States of America

## Abstract

**Background:**

Proper function of the mammalian brain relies on the establishment of highly specific synaptic connections among billions of neurons. To understand how complex neural circuits function, it is crucial to precisely describe neuronal connectivity and the distributions of synapses to and from individual neurons.

**Methods and Findings:**

In this study, we present a new genetic synaptic labeling method that relies on expression of a presynaptic marker, synaptophysin-GFP (Syp-GFP) in individual neurons *in vivo*. We assess the reliability of this method and use it to analyze the spatial patterning of synapses in developing and mature cerebellar granule cells (GCs). In immature GCs, Syp-GFP is distributed in both axonal and dendritic regions. Upon maturation, it becomes strongly enriched in axons. In mature GCs, we analyzed synapses along their ascending segments and parallel fibers. We observe no differences in presynaptic distribution between GCs born at different developmental time points and thus having varied depths of projections in the molecular layer. We found that the mean densities of synapses along the parallel fiber and the ascending segment above the Purkinje cell (PC) layer are statistically indistinguishable, and higher than previous estimates. Interestingly, presynaptic terminals were also found in the ascending segments of GCs below and within the PC layer, with the mean densities two-fold lower than that above the PC layer. The difference in the density of synapses in these parts of the ascending segment likely reflects the regional differences in postsynaptic target cells of GCs.

**Conclusions:**

The ability to visualize synapses of single neurons *in vivo* is valuable for studying synaptogenesis and synaptic plasticity within individual neurons as well as information flow in neural circuits.

## Introduction

A fundamental goal of neuroscience is to describe the structure of neural circuits at the levels of single cells and synapses and to understand how this structure determines nervous system function. The precise pattern of synaptic connections plays an instrumental role in directing the function of a particular circuit to enable information acquisition, processing, storage, and ultimately the control of behavior [1].

Chemical synapses are characterized by specialized subcellular compartments in pre- and post-synaptic neurons. The presynaptic terminal is a specialized subcellular structure with abundant synaptic vesicles containing neurotransmitters as well as an active zone that facilitates vesicle fusion and the release of neurotransmitters into the synaptic cleft. The postsynaptic density, the region of the postsynaptic neuron directly juxtaposed to the presynaptic terminal, contains a high concentration of neurotransmitter receptors, channels, and downstream signaling molecules essential for information transmission across the synapse. Synapses can be clearly identified by electron microscopy (EM) based on the synaptic vesicles and density of accumulated proteins in the pre- and post-synaptic regions, and can be visualized by light microscopy through immunostaining for synaptic components or by using transgenic synaptic components tagged with fluorescent molecules.

The capability to visualize synapses with genetically encoded markers provides a valuable tool for studying synaptogenesis, synaptic plasticity and information flow through neural circuits. However, given the high density of neurons and synapses in the vertebrate CNS, visualizing synapses in all cells at the same time makes it very difficult to discern connectivity patterns. The ability to view a circuit by visualizing synapses only in small subsets of cells can greatly ease the characterization of synapses and their involvement in circuit formation and function. Here, we describe a genetic method that allows labeling of a single neuron or small subsets of neurons with one fluorescent marker while simultaneously labeling their pre-synaptic terminals with a different fluorescent marker. We use the cerebellar granule cell as a model to show the utility of our method in characterizing the organization and development of presynaptic terminals within individual neurons.

## Results and Discussion

### A Genetic Strategy to Label Presynaptic Terminals in Single Neurons

**Figure 1:**
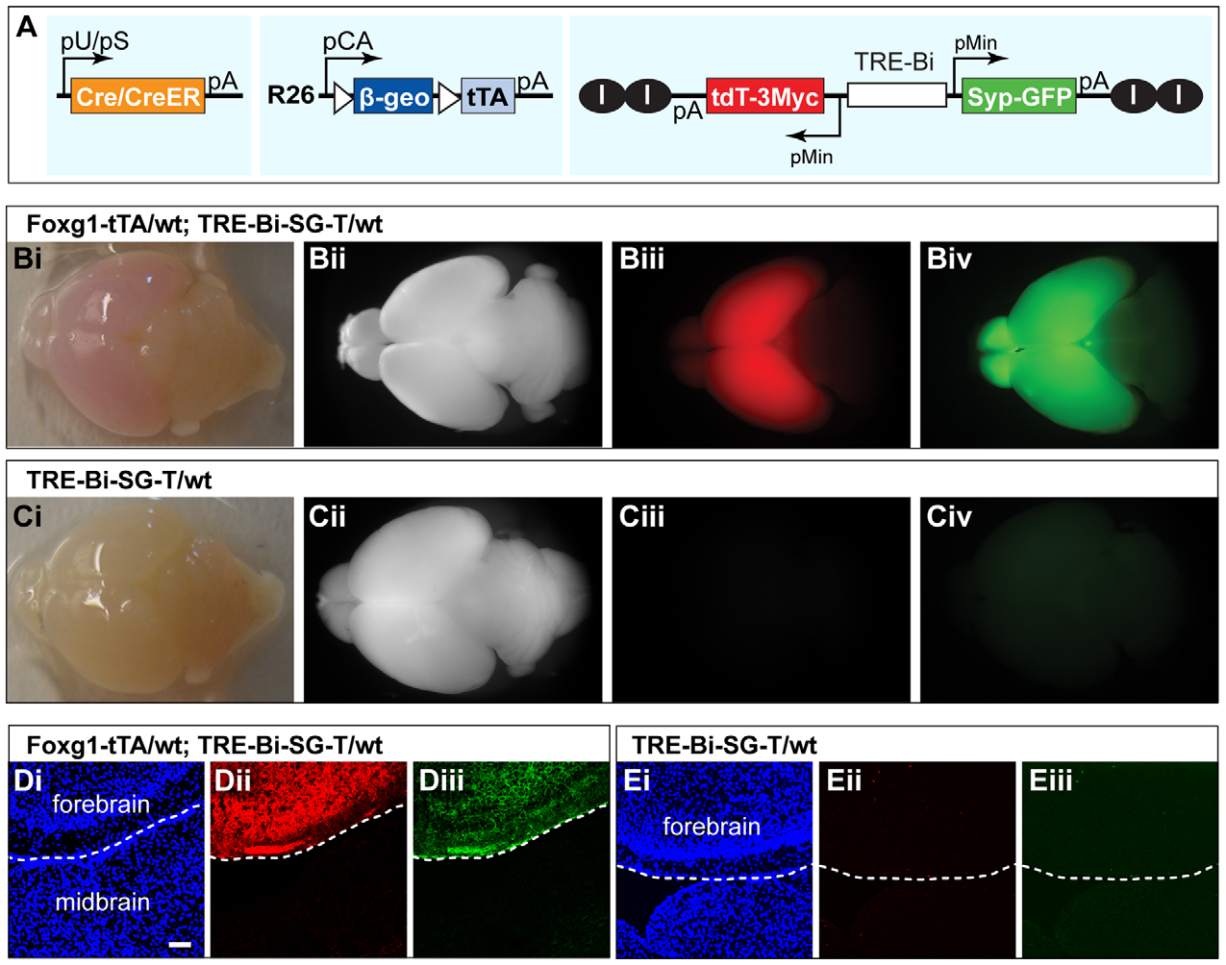
Strategy for creating a transgenic tripartite system for presynaptic labeling *in vivo*. (A) Schematic representation of the three transgenes used for *in vivo* synaptic labeling: A ubiquitous (pU) or tissue-specific (pS) promoter driven Cre/CreER transgene (left); A Cre/CreER-dependent tTA knock-in (ZtTA) targeted to the ROSA26 locus (middle); A bidirectional TRE (TRE-Bi) transgene driving Synaptophysin-GFP (Syp-GFP) and tdT (TRE-Bi-SG-T) (right). I, chicken β-globin insulators were present in one version of this transgene (iiTRE-Bi-SG-Tii). (B–E) Proof-of-principle experiments showing tTA-dependent expression of Syp-GFP and tdT. (B) Whole-mount brain images of a double transgenic mouse (Foxg1-tTA/wt; TRE-Bi-SG-T/wt). (C) Whole mount brain images of a control mouse (TRE-Bi-SG-T/wt). Bii and Cii, bright field images; Biii and Ciii, tdT expression; Biv and Civ, Syp-GFP expression. (D) Confocal image of a brain section from a P7 double transgenic mouse (Foxg1-tTA/wt; TRE-Bi-SG-T/wt) showing that expression of the TRE-Bi transgene is restricted to forebrain. (E) Confocal image of a brain section from a P7 control mouse (TRE-Bi-SG-T/wt) showing that the TRE-Bi transgene alone is not expressed when a tTA transgene is absent. Di and Ei, DAPI; Dii and Eii, tdT; Diii and Eiii, Syp-GFP. White dashed line demarks the forebrain-hindbrain boundary. Scale bar = 50 µm.

To visualize the presynaptic distribution in single neurons within the mouse brain, we designed a strategy in which a tripartite system was used to express fluorescently tagged synaptic proteins with both spatial and temporal control (Figure 1A). We used the Cre/loxP system to define the type and frequency of labeled neurons: Cre or tamoxifen-inducible Cre (CreER) can be driven by a variety of specific promoters in transgenes generated by the mouse community (for example, [2], [3], [4], [5], [6], [7]). In neurons where Cre or CreER is active, a transcriptional stop site flanked by loxP sites preceding the tetracycline transactivator tTA [8] is excised, allowing tTA expression. In turn, tTA drives marker expression under the control of the TRE (tetracycline response element) in front of a minimal promoter.

To create a mouse line with Cre-dependent tTA expression (Figure 1A), we cloned a loxP-flanked β-geo [9] in front of the open reading frame of tTA, and then placed the transcription unit under the control of a ubiquitous promoter (pCA, see Methods for details) [3], [10]. This construct (ZtTA) was targeted to the ROSA26 locus [11] using standard ES cell-based homologous recombination techniques [12], [13], [14]. We chose the ROSA26 locus because it supports global expression in many tissues during mouse development [15], [16]. We generated mouse lines after successful germline transmission of the knock-in transgene.

To achieve tTA-dependent labeling of presynaptic terminals in one color and the entire neuron in another, we placed Synaptophysin (Syp) tagged with green fluorescent protein (GFP) and a red fluorescent protein tdTomato (tdT) [17] under the control of a bidirectional TRE-containing promoter (TRE-Bi, Clontech). This transgene, TRE-Bi-SG-T, should enable labeling of whole neurons in red and presynaptic terminals in green. To enable fluorescent signal enhancement using immunofluorescence from TRE-Bi-SG-T, tdT was tagged with three Myc epitopes at the C-terminus. Syp is an integral synaptic vesicle glycoprotein [18] and is a widely used marker for synapses [19], [20], [21], [22], [23], [24], [25], [26], [27], [28]. Previous studies have shown that Syp-GFP co-localizes with the endogenous synaptic vesicle protein SV2 [23], [24], and that the fusion protein, Syp-GFP, does not alter presynaptic function [21].

One potential caveat of the tetracycline binary expression system is that TRE transgenes might have tTA/rtTA-independent expression due to local enhancer effects at the site of transgene insertion [29]. To prevent possible position effects, we engineered another version of the TRE transgene that is flanked by two copies of the chicken β-globin insulator [30] on each side. Additionally, these insulators block expansion of heterochromatin into the transgene, potentially preventing it from being silenced [31], and they decrease the basal expression and increase inducibility of TREs [32]. The two versions of the TRE-Bi-SG-T construct, with or without insulators, were introduced into mice by standard pronuclear injection to generate a number of founders with randomly integrated transgenes (Figure S1A).

### Characterization of the Transgenes

To test these TRE transgenic lines, we crossed individual founder lines with Foxg1-tTA, which drives TRE transgene expression in the forebrain [33]. None of the TRE transgenic lines exhibited tTA-independent expression in the brain. 5 out of 16 TRE-Bi-SG-T lines exhibited broad, tTA-dependent transgene expression in the forebrain (Figure 1B and D). The remaining 11 lines exhibited no expression, sparse expression or highly restricted expression of the transgene to specific cell types in the forebrain (notably, olfactory receptor neurons and vomeronasal receptor neurons), despite the ubiquity of Foxg1-tTA in the forebrain. Insulators appear to have a beneficial effect on the probability and broadness of TRE transgene expression (Figure S1A). We focused on the two TRE-Bi-SG-T lines with broadest and strongest expression, one of which carries a single copy of the transgene without insulators, and the other of which carries 3 copies of the transgene with insulators (Figure S1B).

**Figure 2:**
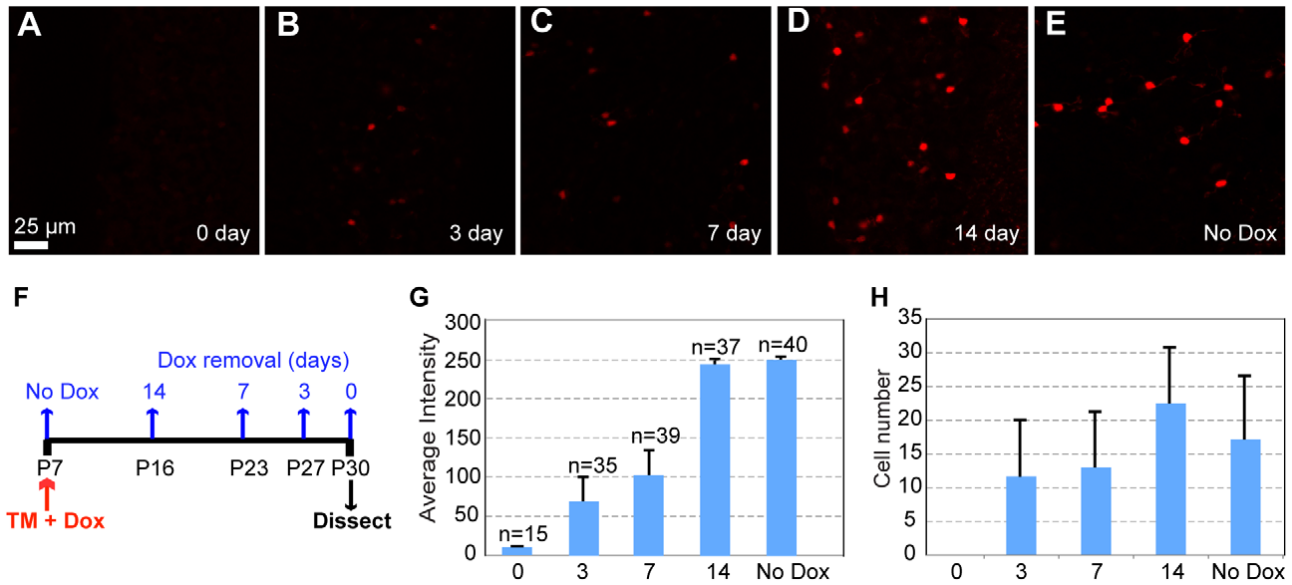
Dox-dependent gene activation. (A–E) Representative confocal images of labeled cerebellar GCs from P30 triple transgenic mouse (ZtTA/wt; TRE-Bi-SG-T/wt; β-actin-CreER/wt). Tamoxifen was injected at P7 and Dox was never administered (E) or it was removed for 0 (A), 3 (B), 7 (C) or 14 (D) days. Scale bar = 25 µm. (F) Diagram of the experimental procedure described above. (G) Quantification of the average fluorescence intensity in cerebellar GC bodies indicates that the expression level of the TRE-driven transgene increases the earlier Dox is removed, reaching maximal expression by 14 days after Dox removal. Error bars represent standard deviation. “n” represents the number of analyzed cell bodies for each condition indicated. Two animals per Dox condition were used for analysis. (H) Quantification of the total number of labeled cerebellar GCs per 225×225 µm^2^ for each Dox condition shows no statistically significant differences between different conditions. Error bars represent standard deviation.

To test the tripartite system, we generated mice that contain 3 transgenes: ZtTA, TRE-Bi-SG-T, and Cre or CreER. We observed labeling in different types of neurons and brain regions depending on the pattern of Cre/CreER transgene expression (data not shown). To suppress marker expression until desired time points, we used Doxycycline (Dox). Dox administration could circumvent potential side effects of synaptic protein overexpression on synapse development. Derepression of TREs should occur upon Dox removal and is dependent on the rate of Dox clearance. In adult mice, transgene induction can take from 24 hours to 1 week, depending on the Dox concentration used [34]. We tested the suppression of transgene expression at different concentrations of Dox (2 mg/ml and 10 µg/ml). No tdT fluorescence signal is detectable upon continuous treatment with either 2 mg/ml (data not shown) or 10 µg/ml Dox in drinking water (Figure 2A). To enable faster TRE transgene reactivation upon Dox removal, we performed all subsequent experiments using the lower Dox concentration.

To study the time course of re-activation of the TRE transgene upon Dox removal, we analyzed the brains at postnatal day (P) 30 for mice that: 1) had never been treated with Dox (no Dox); 2) had been continually treated with Dox from tamoxifen injection (P7) until dissection (0 days of Dox removal); or 3) had been treated with Dox from tamoxifen injection (P7) until 14, 7 and 3 days before dissection (Figure 2F). The tdT fluorescence can be detected after a single day of Dox removal using immunohistochemistry (data not shown), and weak tdT fluorescence can be detected without immunostaining 3 days after Dox removal (Figure 2B). Nascent tdT fluorescence signal increased with time following Dox removal (Figure 2A–E). We quantified Dox-dependent regulation by measuring the fluorescence intensity of tdT in labeled cerebellar GC bodies, and found that tdT intensity increases steadily with time (Figure 2G), reaching full expression 14 days after Dox removal (Figure 2G, compare ‘14’ and ‘No Dox’). We also quantified the number of labeled cells under these different conditions (except for the condition where Dox is always present, as we could not detect tdT signal in this case) and did not observe a statistically significant difference between different conditions (Figure 2H). These experiments clearly demonstrate that our marker expression can be regulated by Dox application.

### Validation of the Synaptic Labeling Tool

**Figure 3:**
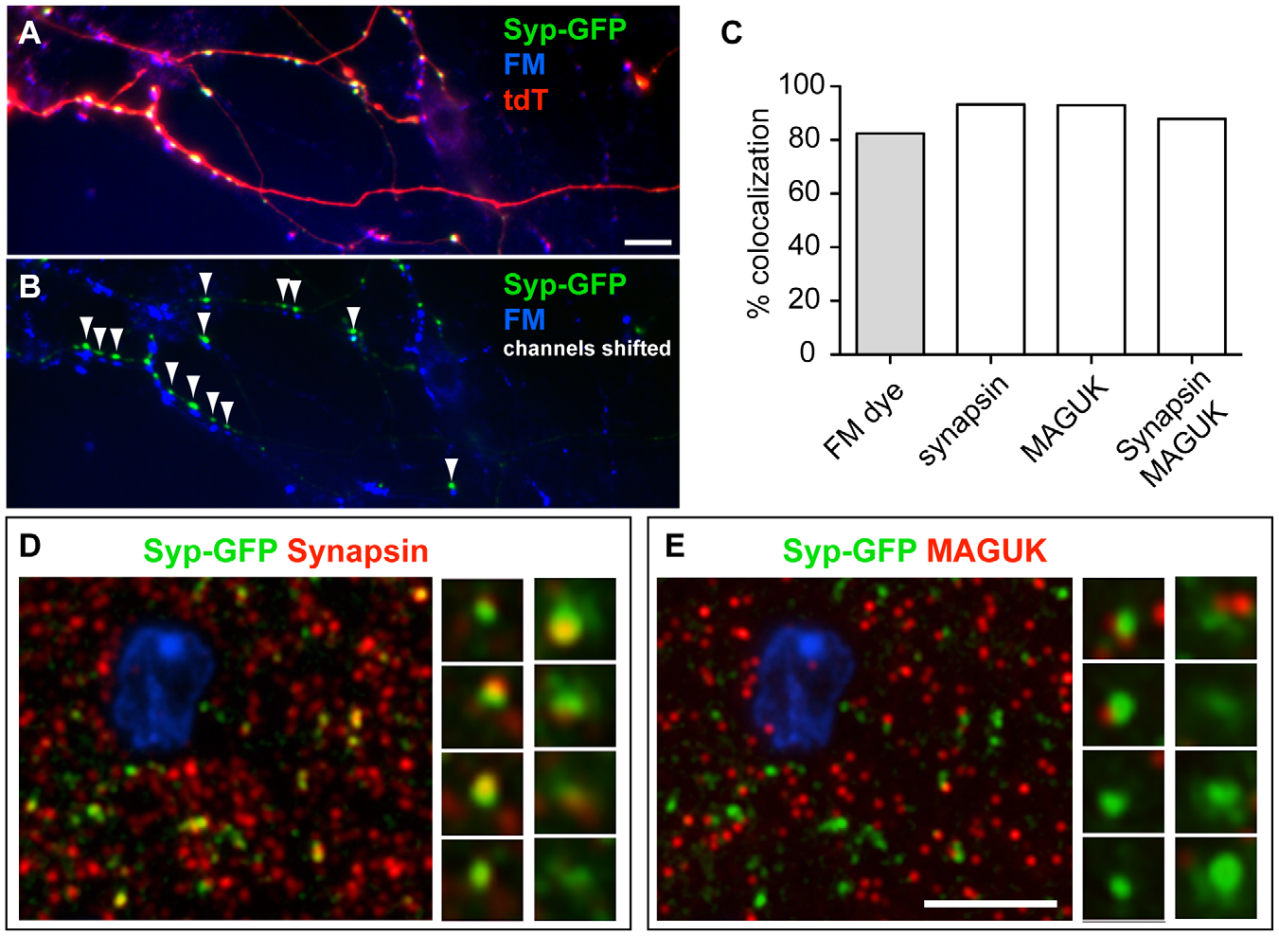
Validation of the synaptic labeling tool. (A) A representative image showing functional presynaptic terminals labeled by FM dye staining (blue) in 14 DIV (days in vitro) dissociated primary hippocampal neurons generated from a P0 triple transgenic mouse pup (ZtTA/wt; TRE-Bi-SG-T/wt; Nestin-Cre/wt). The presynaptic termini, labeled by Syp-GFP (green) and the FM dye (blue) are located on axonal processes (red) and therefore appear white or yellowish. (B) The Syp-GFP and FM-dye channels from panel A have been shifted to show that most Syp-GFP puncta (green, white arrowheads) located along the processes colocalize with FM dye (blue) puncta. Scale bar is 5 µm. (C) Quantification of percentage of Syp-GFP colocalized with FM dye (Grey bar for FM dye experiment in A–B), synapsin and MAGUK (for array tomography experiment in D–E). The total number of counted Syp-GFP puncta is 1176 and 314, for the FM dye experiment and array tomography experiment, respectively. Two animals were used for the FM dye experiment. (D–E) Representative array tomography images from a P35 triple transgenic mouse brain (ZtTA/wt; TRE-Bi-SG-T/wt; β-actin-CreER/wt) with tamoxifen administered at E9.5 showing *in vivo* presynaptic localization of Syp-GFP puncta. Left panels represent images from a single ultrathin (70 nm) section showing colocalization of Syp-GFP (green) with synapsin (red, C) or MAGUK (red, D). Right panels show two examples (each example in a single column) of 4 serial sections through a single Syp-GFP punctum.

To validate that our Syp-GFP labels *bona fide* presynaptic terminals, we used both an FM dye uptake assay in cultured neurons [35] and array tomography *in vivo* [36]. FM dye is a lipophilic molecule that exhibits low fluorescence in water but fluoresces intensely upon binding the outer layer of the plasma membrane; it can be internalized via synaptic vesicle recycling after neuronal excitation [37], [38]. Only active synapses are labeled after neuronal excitation, and thus colocalization between the sites of FM dye uptake and synaptic markers can provide a quantitative characterization of the veracity of a synaptic marker. To examine the extent of this colocalization, we performed an FM dye uptake assay on primary hippocampal neuronal cultures from triple transgenic mice with the genotype ZtTA/wt; TRE-Bi-SG-T/wt; Nestin-Cre/wt [39]. Neurons were excited using an established maximal stimulation protocol (1200 action potentials at 10 Hz) to label the total recycling vesicle pool [40]. We then determined the degree of colocalization between FM-positive puncta and GFP-positive puncta. We found that ∼85% of GFP-positive puncta were also labeled by FM dye (Figure 3A–C), indicating functional presynaptic terminals. Our results are consistent with previous reports demonstrating that the number of FM-positive puncta that correlate with structurally identified synapses range from 46% to 88% depending on the action potential triggering protocol [35]. This finding suggests that the vast majority of Syp-GFP puncta labeled by our genetic strategy are *bona fide* presynaptic terminals.

Array tomography is a recently developed imaging method in which immunofluorescence labeling is performed on ordered, serially collected, ultra-thin (50–200 nm), resin-embedded sections [36]. It allows quantitative, high-resolution, large-field volumetric imaging of a number of antigens, fluorescent proteins, and ultrastructure in individual tissue specimens. Here we used array tomography to further determine the extent to which our presynaptic markers are localized to synapses *in vivo*. We examined a triple transgenic mouse with the genotype of ZtTA/wt; TRE-Bi-SG-T/wt; Nestin-CreER.line5/wt [41] at P35 with 2 mg of tamoxifen injected at embryonic day (E)9.5. We chose the molecular layer of the cerebellum for array tomography due to the abundance of labeled synapses belonging to the cerebellar GCs in this region. We quantified the number of Syp-GFP puncta that overlap with immunofluorescent puncta of endogenous Synapsin (a presynaptic marker) or that are adjacent to immunofluorescent puncta of MAGUK (a postsynaptic marker) (Figure 3D–E). Out of 314 Syp-GFP puncta counted, 276 overlapped with Synapsin and were adjacent to MAGUK, 17 overlapped only with Synapsin, 16 were adjacent only to MAGUK, and 5 had none of the synaptic proteins nearby. Thus, 98% of Syp-GFP puncta colocalized with at least one of the endogenous synaptic markers, and 88% of Syp-GFP puncta colocalized with both endogenous synaptic markers (Figure 3C), suggesting that Syp-GFP labels *bona fide* presynaptic terminals *in vivo*.

### Visualization of Presynaptic Terminals in Cerebellar Granule Cells *In Vivo*

**Figure 4:**
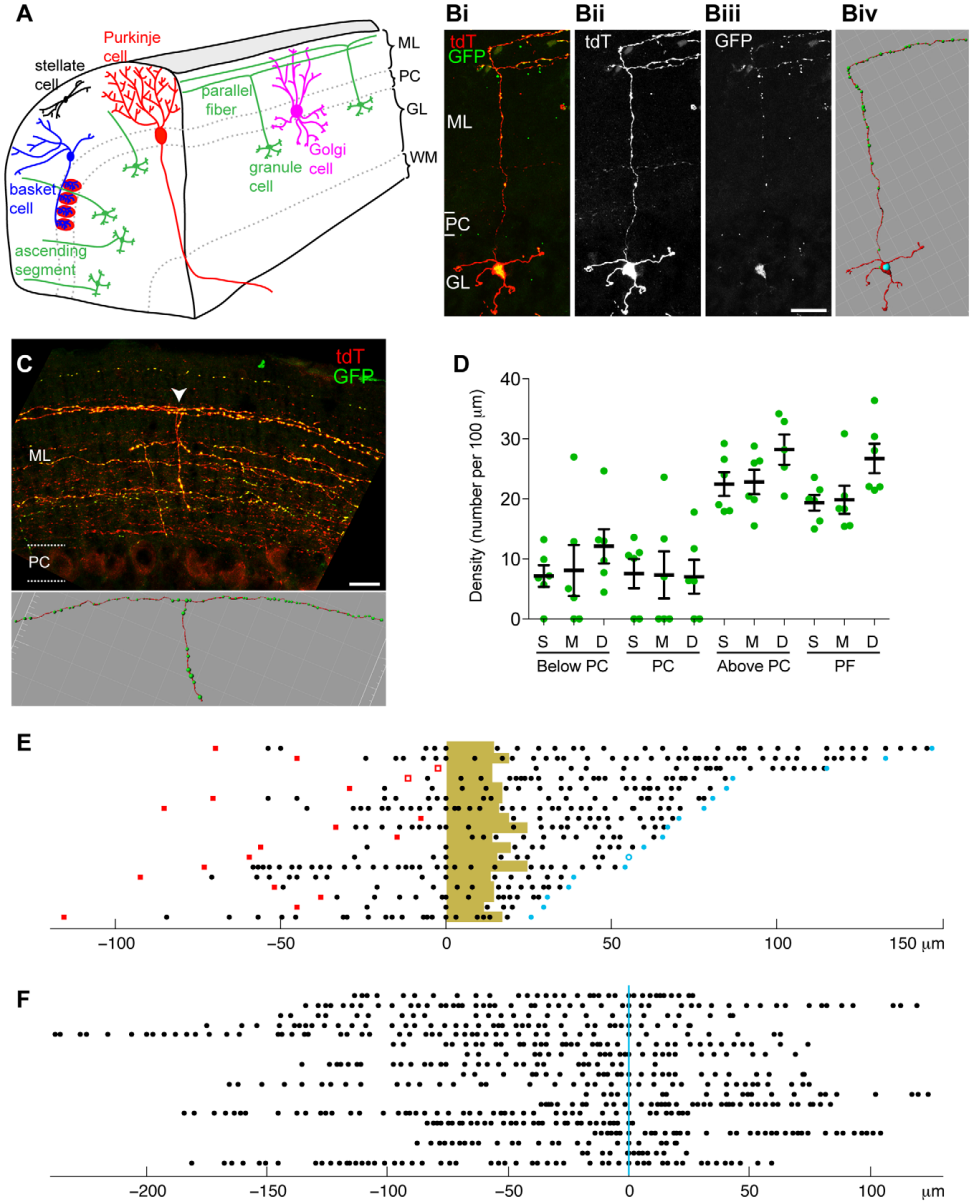
Quantitative analysis of Syp-GFP distribution in cerebellar granule cells. (A) Schematic illustration of the organization and major neuronal types of the cerebellar cortex. ML, molecular layer; PC, Purkinje cell body layer; GL, granular layer; WM, white matter. (B) Representative confocal image of a labeled mature cerebellar GC in a ZtTA/wt; TRE-Bi-SG-T/wt; β-actin-CreER/wt mouse with tamoxifen administered at P7 and dissected at P30, 7 days after Dox removal. The overlap between Syp-GFP puncta and tdT labeled GC processes is evident in the merged image, where Syp-GFP puncta appear yellow. Cerebellar layers (as illustrated in panel A) are marked with white lines. Scale bar = 20 µm. The last panel shows a snapshot of the same cerebellar GC after filament and puncta tracing in Imaris. (C) Representative confocal image of labeled parallel fibers and a snapshot of a single traced parallel fiber (bifurcation point marked by a white arrow in the upper image). (D) Quantification of Syp-GFP mean density (the number of puncta per 100 µm) for different populations of GCs (N = 6 for each population, except N = 5 for the deep sublayer ascending segment above PC layer). We quantified the density of Syp-GFP puncta in the ascending segment below the PC layer (Below PC), within the PC layer (PC), above the PC layer (Above PC) and in the parallel fibers (PF) for GCs projecting their axons to superficial (S), middle (M) or deep (D) regions of the molecular layer. Each green dot represents a data point. A black line marks the mean of each column. Error bars are ± standard error of the mean (SEM). Six animals were used for analysis. (E) Plot showing the Syp-GFP puncta distribution along linearized ascending segments of each traced GC. The light brown shading represents the Purkinje cell layer. Red solid squares on the left represent the locations where individual ascending axons initiate from corresponding cell bodies. Red empty squares represent the locations where an ascending axon initiates from a dendrite. The open blue circle on the right represents the bifurcation point without a Syp-GFP punctum. Blue dots on the right represent the bifurcation points containing a Syp-GFP punctum. Numbers below the plots represent distance in µm. Cells are aligned where the PC layer starts; to the left below the PC layer, and to the right above the PC layer. (F) A plot showing the distribution of Syp-GFP puncta along linearized parallel fibers of traced GCs. The blue vertical line marks the bifurcation points. Numbers below the plots represent distance in µm. Axons are aligned at the bifurcation point.

Having validated our synaptic labeling tool, we used it to examine the spatial patterns of presynaptic terminals in single neurons *in vivo*. We focused on the cerebellar granule cells (GCs), the most abundant neurons in the mammalian brain, as model neurons for our study. The organization and diverse cell types of the cerebellum are diagrammed in Figure 4A. Cell bodies of the GCs are located in the granular layer of the cerebellar cortex. Each GC sends out 4–5 dendritic branches, which end in claw-like varicosities where they form synapses with axon terminals of mossy fibers, the major presynaptic partners of GCs. Each GC sends an ascending axon, which travels towards the pial surface of the cerebellar cortex by traversing the granular layer, Purkinje cell layer and part of the molecular layer. Each ascending axon terminates at a specific depth of the molecular layer according to the timing of GC differentiation and bifurcates into a parallel fiber that runs parallel to the pial surface and intersects perpendicularly the planar dendritic trees of numerous Purkinje cells, which are the major postsynaptic partners of GCs (Figure 4A) [42], [43], [44], [45]. Thus, the GC axon can be divided into two discrete parts: the ascending segment and the parallel fiber. In addition to providing major synaptic input to Purkinje cells, GCs are also known to provide synaptic input to local inhibitory neurons including basket cells, stellate cells and Golgi cells (Figure 4A) [46], [47].

Using our genetic strategy to label individual mature GCs, we found that the red fluorescent marker, tdT, strongly labels the cell body, axon and dendrites (Figure 4B–C). In contrast, Syp-GFP is localized to puncta along the ascending segment and the parallel fiber (Figure 4B–C), reflecting synaptic connections with Purkinje cells and local inhibitory interneurons. We also observed Syp-GFP signal in the dendritic region, although this signal was more diffuse and not discretely punctate (see below). The cell body was also strongly labeled by Syp-GFP fluorescence, likely reflecting the soma as the site of protein synthesis.

### Distribution of Presynaptic Terminals along Different Segments of Granule Cell Axons

Anatomically, GC synapses are located on the ascending segment or the parallel fiber. Whether these two anatomically distinct inputs play different functional roles is a controversial and frequently debated topic [48], [49], [50], [51], [52], [53], [54]. Despite early EM studies that identified synapses in GC ascending segments [47], their presence has not been fully addressed in many descriptions of cerebellar circuitry [42], [55], [56]. Historically, the parallel fiber synaptic inputs have been considered to be the primary excitatory route for Purkinje cells; indeed, previous anatomical data indicate that most of the synapses between the GCs and Purkinje cells are located on GC parallel fibers. However, estimates of the percentage of synapses located on GC ascending segments vary between 3 and 20% [57], [58], [59], [60], [61]. Recent evidence suggests that the synapses along the ascending segment may carry as much or more synaptic weight than the connections on the parallel fiber and hence have an important functional role during cerebellar cortical signaling and processing [49], [50], [57]. Therefore, we wanted to utilize our synaptic labeling tool to further investigate the subcellular distribution of GC presynaptic terminals.

To examine the distribution and density of presynaptic puncta in GCs, we took advantage of the sparse labeling offered by our method and used 3-D image rendering to determine the location of the puncta and the intervals between puncta (Figure 4B–C). This analysis should provide more accurate measurements compared to traditional methods using 2-D measurements and statistical estimation [59], [60], [62]. We linearized each traced GC ascending segment (Figure 4E) and plotted the position of Syp-GFP puncta along the line (black dots). We noted the beginning of each ascending segment (red squares) and the bifurcation point of each parallel fiber (blue dots; solid dot represents the Syp-GFP punctum at the bifurcation point). We also marked the boundaries of the PC layer (Figure 4E, yellow highlight). As can be seen from such plots, Syp-GFP puncta are distributed along the entire ascending segment: within the granular layer, the PC layer and the molecular layer. We calculated the mean density of Syp-GFP in the ascending segment for each layer (Figure 4D). We found no significant difference between the density of presynaptic puncta in the ascending segment below the PC layer and within the PC layer (Kruskal-Wallis, p = .0578). The presynaptic density in the ascending segment above the PC layer is significantly (about two-fold) higher than below or within the PC layer (Kruskal-Wallis, p = .0002). We also note that the coefficients of variation for presynaptic density are higher in the regions of the ascending segment below and within the PC layer than in the regions above the PC layer (Figure S2A). Finally, we note that the inter-puncta intervals are distributed randomly (Figure S2B–D), suggesting that post-synaptic targets are not clustered, nor is there an intrinsic mechanism to space synapses evenly along the GC axon.

Due to the differential dendritic distribution of GC postsynaptic partners (Figure 4A), presynaptic terminals in different parts of the GC ascending segments likely connect to different types of cells and may therefore have different functional outputs. Specifically, dendrites of Purkinje cells, basket cells and stellate cells are all restricted to the molecular layer, so GCs can connect to them only through synapses along the ascending segments and parallel fibers within the molecular layer. On the other hand, Golgi cell dendrites begin within the granular layer and extend into the molecular layer and can therefore receive presynaptic input from GCs both within the granular layer and in the molecular layer. Indeed, EM studies have previously identified synapses between GCs and Golgi cells both in the granular layer and in the molecular layer [47]. GC input to Golgi cells serves a different role than their input to the other two types of local interneurons, stellate and basket cells. The major postsynaptic targets of stellate and basket cells are Purkinje cells, so GC input to these cells serves as feed-forward inhibition. The major targets of Golgi cells are the GCs themselves, so GC input to Golgi cells serves as feedback inhibition. Our finding together with previous EM studies [47] suggests that in addition to the canonical cerebellar circuit where parallel fibers activate Golgi cells, GC ascending segments might provide substantial input to Golgi cells via synapses within the granular layer. Since these synapses are located closer to the soma of Golgi cells, this feedback mechanism might be more powerful than the one originating from GC-Golgi synapses within the molecular layer.

We also characterized the distribution of synapses formed on the GC parallel fibers. For each parallel fiber, we plotted the bifurcation point and the Syp-GFP puncta after linearizing the 3-D tracing (Figure 4F). The Syp-GFP puncta are distributed randomly, without any obvious pattern, along the parallel fibers (Figure S2D). We also calculated the mean density of Syp-GFP puncta on parallel fibers and compared it to the mean density from the three regions of the ascending segment. Syp-GFP density in the parallel fibers is not significantly different from that in the ascending segments above the PC layer (Kruskal-Wallis, p = .7867), but is significantly different from the density in the ascending segments below and within the PC layer (Kruskal-Wallis, p = .0004). Moreover, the coefficients of variation for presynaptic densities are more similar between ascending segment above the PC layer and the parallel fiber then between the ascending segment above the PC layer and within/below the PC layer (Figure S2A). Due to technical limitations, we could not trace parallel fibers to their ends, and thus our results (Figure 4F) reflect only the synaptic distribution within the proximal regions of parallel fibers. As synapses on the ascending segments above the PC layer and on parallel fibers form mostly between GCs and Purkinje cells, the synapses on these two portions of GC axons might be functionally comparable. These findings further support the hypothesis that the difference in synaptic density in the three regions of the ascending segment is due to the change in the postsynaptic target cell population.

In the molecular layer, our data show that on average 25 presynaptic terminals are found per 100 µm along the ascending segments and the parallel fibers, or one presynaptic terminal for every 4 µm (Figure 4D). This density is considerably higher than previous estimates of one presynaptic terminal for every 6.8 µm or 9.4 µm along the parallel fibers or ascending segments, respectively, based on bouton counting along Golgi-stained granule cell axons in mice [60]. A likely explanation for this difference is that our tracing method offers more sensitive detection of presynaptic terminals compared to previously used methods that rely on counting varicosities along axons as synapses [60]. Our finding implies that granule cells make denser connections in the molecular layer than previously thought. A recent study using calcium imaging suggests that the width of planar Purkinje cell dendritic tree is on average 7 µm [63]. Given that each parallel fiber usually forms 1–2 synapses with a Purkinje cell [42], it is possible that a parallel fiber synapses with almost all Purkinje cells whose dendritic branches are within its reach.

### Granule Cell Presynaptic Density Does Not Vary in Sublayers of the Molecular Layer

We have previously reported that most GCs stack their axons within the molecular layer in a temporal sequence: GCs that differentiate first leave their axons in the deepest sublayer in the molecular layer, whereas GCs that differentiate progressively later leave their axons in progressively more superficial sublayers [45]. To investigate if differences in synaptic marker distribution exist between these different sublayers, we arbitrarily divided the molecular layer into 3 equally thick sublayers: deep, middle and superficial. Examination of multiple single GCs from each sublayer did not reveal a stereotyped localization pattern of Syp-GFP presynaptic puncta to any particular region of the ascending segments or parallel fibers (Figure 4E–F). There is no statistically significant difference in presynaptic densities between GCs projecting to different sublayers in the ascending segment below (Kruskal-Wallis, p = .3296), within (Kruskal-Wallis, p = .9575) or above (Kruskal-Wallis, p = .2350) the PC layer (Figure 4D). Parallel fibers in the deep sublayer appear to have a slightly higher presynaptic density than parallel fibers in other two sublayers, although this difference does not reach statistical significance (Kruskal-Wallis, p = .0504; Figure 4D). Thus, based on synaptic labeling, GCs projecting to different sublayers of the molecular layer exhibit similar overall synaptic marker distributions.

### Granule Cell Presynaptic Distribution Does Not Vary with the Duration of Syp-GFP Expression

We wanted to examine if different Dox treatment conditions influenced synaptic distribution. The synaptic distribution plots (Figure 4E–F) were collected from triple transgenic mice that have undergone different Dox treatments, ranging from no Dox to Dox treatment until 7 days before examination (Figure S3). We compared Dox treated and untreated samples, but did not observe significant differences between synaptic densities in any region of the GC (Mann Whitney; below PC, p = .3726; PC, p = .4121; above PC, p = .7921; PF, p = .3311). We also did not observe notable differences in the coefficient of variation between Dox treated and untreated samples (Figure S3). Together with previous observations that overexpression of Syp or Syp-GFP does not compromise presynaptic function [21], we conclude that neurons labeled by our tool likely develop and function normally.

### Maturation of Presynaptic Marker Distribution during Development

**Figure 5:**
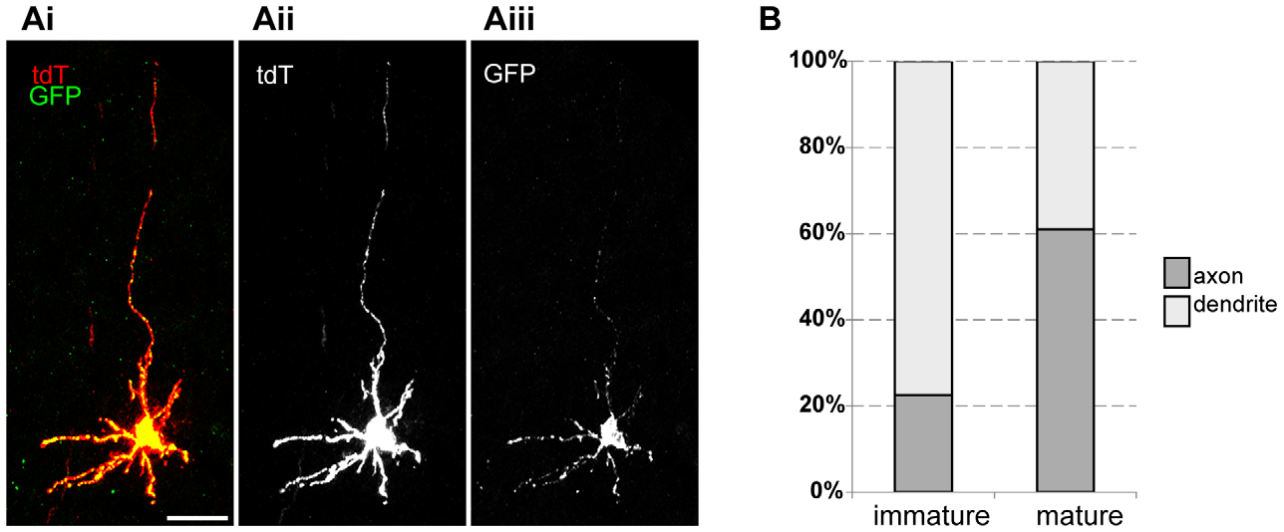
Syp-GFP distribution patterns in developing and mature GCs. (A) Representative confocal image of a labeled immature cerebellar GC from the mouse with the following genotype: ZtTA/wt; TRE-Bi-SG-T/wt; β-actin-CreER/wt, with tamoxifen administered at P7, and dissected at P15. In the merged panel Syp-GFP appears yellow, while tdT (red) fills the processes. (B) Quantification of Syp-GFP distribution in axonal and dendritic regions in mature and immature GCs showing that Syp-GFP becomes enriched in the axon after maturation. The graph shows an average for 10 cells for each condition. For mature cells, the standard deviation is ±11%; for immature cells, the standard deviation is ±7%. Two animals were used for analysis.

The ability to visualize Syp-GFP puncta within single neurons also provided a means to study the distribution of presynaptic markers during neuronal maturation. In mice, GCs are born during the first three postnatal weeks. At any time during this period, different GCs are in different maturation stages depending on their birth timing (Espinosa and Luo, 2008). When we visualized sparsely labeled GCs at P15, we could unambiguously distinguish both mature and immature GCs. Figure 5A shows a typical example of a labeled single immature GC at P15. Immature GC has more than 4 or 5 dendritic processes and compared to those of the mature GC, these dendrites are thinner and lack the terminal dendritic claws. We found that Syp-GFP fluorescence can be detected in all the processes in immature GCs, including both the potential axon and dendrites (Figure 5A). As the neurons mature, Syp-GFP puncta are localized more specifically to the axon (Figure 4B), although we still observe a couple of distinct puncta and diffuse Syp-GFP signal in the dendritic claws of some cells.

To quantify the changes in Syp-GFP subcellular distribution during GC maturation, we analyzed the percentage of Syp-GFP localized to the axonal region versus the dendritic region for mature and immature GCs. This quantitative analysis excluded the cell body to remove the confounding variable of recently synthesized Syp-GFP. For immature GCs, we denoted the single, longest, thin ascending process as the axon, and the other short, thick, radiating processes as dendrites. Figure 5B shows that in mature GCs, 61±11% of Syp-GFP is in the axons and 39±11% is in the dendrites. In immature cells, 22±7% of Syp-GFP is in the axons and 78±7% is in the dendrites. Therefore, we conclude that Syp-GFP is enriched in axons after maturation and after neuronal polarity is established.

Previous studies in dissociated hippocampal neurons in culture showed that VAMP2 is initially present in both the axon and dendrites before being enriched in the axon [64], [65]. This observation of “mistargeted” synaptic vesicle protein and subsequent targeting to the correct compartment has been suggested as a general developmental tenet for some synaptic components [64], [65]. Our observation of Syp-GFP in immature and mature GCs *in vivo* further supports the developmental redistribution of presynaptic terminal markers between the dendritic and axonal compartments. However, it is important to point out that even in mature granule cells, a substantial fraction of Syp-GFP is distributed in dendrites. The dendritic localization of Syp-GFP may be caused by overexpression of this marker in our method, although endogenous synaptophysin has also been observed in dendrites by array tomography studies (KDM & SJS, submitted).

### Distribution of Presynaptic Terminals in Other Neuronal Types

**Figure 6:**
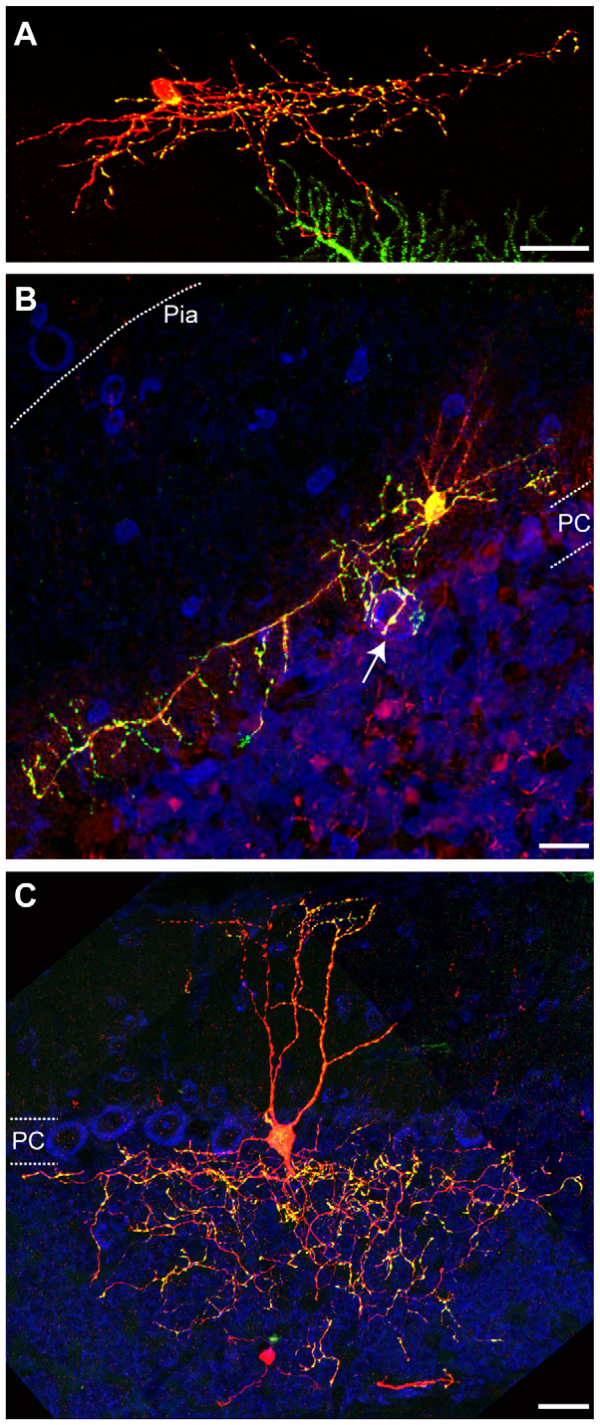
Morphology and presynaptic distribution in cerebellar interneurons. Representative images of Syp-GFP distribution in a cerebellar stellate cell (A), basket cell (B) and Golgi cell (C). Syp-GFP (green), tdT (red), TOTO3 (blue). White dashed lines (B) highlight the PC layer (estimated by the TOTO3 signal of PC bodies) and the pial surface. Arrow points to a “basket.” Labeled GC axons in (B) were removed for presentation purposes using Photoshop. White dashed lines (C) highlight the PC layer in the cerebellum (estimated by the TOTO3 signal of PC bodies). All cells are from triple transgenic mice (ZtTA/wt; TRE-Bi-SG-T/wt; β-actin-CreER/wt) dissected at P21 (A), P30 (B) and P15 (C). Tamoxifen was administered at E9.5 (A), P7 (B) and E17.5 (C). Scale bars are 20 µm.

Finally, we present examples of presynaptic terminal labeling in other neuronal types in the cerebellar cortex. The cerebellar stellate cell body lies in the molecular layer and its axon projects a considerable distance towards the surface of a folium, giving off numerous descending and a few ascending collaterals in its initial portion [43], [47]. Figure 6A shows a partially reconstructed stellate cell labeled by our genetic strategy. The basket cell sends its axon tangentially across the Purkinje cell body layer and issues descending branches to surround Purkinje cell bodies forming “baskets” [43], [47]. Figure 6B shows a partially reconstructed basket cell with an arrow marking a “basket.” We observe that in both the stellate and basket cells, Syp-GFP puncta are mainly located on the thinner processes (presumptive axons) and not on the thicker branches (dendrites). For basket cells, Syp-GFP puncta are particularly enriched in terminal branches that surround Purkinje cell bodies. Golgi cells are located in the upper portion of the granular layer and send dendrites to the molecular layer while their axons ramify within the granular layer [43], [47]. Figure 6C shows a labeled cerebellar Golgi cell at P15. Its cell body lies near Purkinje cell bodies; its descending axon branches meander through a broad region of the granular layer, giving rise to an extensive arborization. There are two thick dendritic processes that arise from the cell body, branch at several points and extend through the molecular layer until the pial surface. Similar to our observation in granule cells, Syp-GFP puncta are mostly enriched in Golgi cell axons in the granular layer, although some Syp-GFP is also present in distal dendritic branches.

In summary, we were able to observe synaptic labeling in distinct types of neurons in the cerebellum and throughout the brain (data not shown), suggesting that our tool can be used to study the synaptic distribution in most neurons in the brain. However, there are exceptions. In the cerebellar cortex, Purkinje cells are poorly labeled by the cytoplasmic tdT marker, but we observe strong dendrite labeling by the Syp-GFP marker (see Figure 6A, green labeling at the bottom). The reason for this aberrant expression behavior is presently unknown. In addition, our analysis of additional cell-types was limited by the use of the ubiquitous β-actin-CreER [5], as it is one of the few well-characterized CreER lines available. In our experience, when this line is used, GC labeling predominates, as GCs are the most abundant neurons in the brain. The labeling of other neuronal types is relatively rare, and single neuron reconstructions are often obstructed by concomitant labeling of more abundant neurons. Another limitation in reconstructing the morphology of complex neurons and their axonal projections is the difficulty to accurately trace individually labeled neurons through multiple tissue sections, as exemplified by our difficulty to trace the entire parallel fibers for GCs (Figure 4F) or entire dendritic and axonal projection patterns for cerebellar interneurons (Figure 6). However, the progressive availability of neuronal type-specific CreER lines and the combination of genetic tools with serial optic sectioning and newly developed reconstruction methods for imaging (Denk and Horstmann, 2004; Micheva and Smith, 2007) should allow specific and reliable labeling and analysis of additional neuronal types.

### Conclusion

In this study, we present a tripartite system to noninvasively label entire processes and presynaptic compartments of sparsely labeled individual neurons with temporal and spatial control. Our genetic method has several advantages compared to other methods, such as viral transduction or *in utero* electroporation, which can in principle achieve similar labeling of both the entire neuron and presynaptic terminals. First, our method has better control over the types of labeled cells by using a specific CreER. Second, we have better control of labeling frequency through the administration of tamoxifen. Third, our method permits the temporal regulation of synaptic marker expression, which can bypass potential developmental defects in synaptic development caused by overexpression of synaptic markers. However, our quantitative analysis in granule cells suggests that changing the duration of Syp-GFP expression has no detectable effect on synapse density and distribution. This finding is consistent with previous findings that Syp-GFP does not interfere with synapse development and function *in vitro* [21]. Fourth, our technique can achieve more consistency in the expression level of marker transgenes between different experiments. Lastly, our method can be combined with additional genetic manipulations, such as the inclusion of other TRE-driven transgenes, which can be temporally controlled with Dox. A potential limitation of our method is that Syp-GFP is also found in cell bodies and to some extent in dendrites in mature neurons. This limitation does not apply to neurons whose axons can be clearly distinguished from dendrites based on their thickness and branching pattern (such as cerebellar granule cells), for which presynaptic terminal distribution can be readily determined based on punctate Syp-GFP expression in the axon compartment.

We used the cerebellar GC to demonstrate the utility of this tool in examining synapse formation during development and synapse distribution in the adult. Compared to previous light microscopy [59], [60] and EM studies [47], our labeling technique provides more sensitive and efficient detection for the study of the distribution of presynaptic compartments. Previous analyses of GCs identified synapses by morphology, namely by synaptic varicosities along the axon [60]. In those cases, the synapses that do not form clearly identifiable morphological varicosities will not be counted. EM studies, while very precise in synapse identification, are limited by the intensity of labor required to collect large volumes of sample. Our method directly visualizes synapses, instead of relying on morphological indicators, and allows for rapid tissue processing. The presynaptic density in granule cell axons in the molecular layer calculated using our method is higher than in previous estimates [60]. Moreover, the finding that GCs possess a significant number of presynaptic terminals along the ascending fibers, especially prior to entering the molecular layer, should be taken into account in future physiological and modeling studies of the cerebellar circuit.

## Materials and Methods

### Ethics Statement

All animal procedures were in compliance with animal care guidelines and were approved by Stanford University's Administrative Panels on Laboratory Care (A-PLAC). Approval number is 14007.

### Recombinant DNA

#### pROSA26-ZtTA

To create a Cre-dependent tTA construct (pCA-ZtTA) we performed a 3-way ligation using the following fragments: 1) The tTA [8] open reading frame with a Kozak sequence (CCGCCACC) was generated by PCR with a pair of primers (5′-AAACTCGAGCCG CCACCATGTCTAGATT-3′ and 5′-TAGAATTCGGGGATCCTC G-3′), followed by XhoI and EcoRI restriction digestion; 2) The loxP-flanked βgeo (a lacZ-neomycin phosphotransferase fusion) coding sequence was obtained from the Z/EG plasmid [9] by restriction digestion with XbaI, subsequent treatment with Klenow to fill-in the 5′ overhangs, and then digestion with XhoI; 3) The expression vector pCA-HZ2 [3] containing a chicken β-actin promoter with CMV enhancer [10] and an SV40 T-antigen poly(A) signal was digested with SmaI and EcoRI. After ligation, the resulting pCA-ZtTA vector was sequenced, cut with PacI and AscI, and subcloned into the pROSA26-PA targeting vector [66] to generate the final targeting construct pROSA26-ZtTA. Before ES cell injection, the functionality of this vector was tested by Western blotting of lysates of COS cells transiently transfected with pROSA26-ZtTA, a TRE-GFP reporter and a Cre plasmid. Only when all three plasmids were co-transfected, we observed high GFP expression (data not shown).

#### pTRE-Bi-SG-T and piiTRE-Bi-SG-Tii

We generated pTRE-Bi-SG-T by a 3-way ligation using the following fragments: 1) A GFP-tagged mouse Synaptophysin (Syp) containing plasmid (gift from Shasta Sabo, Case Western Reserve University) was digested with NheI and BssSI, generating a fragment containing full-length Syp fused to a portion of GFP; 2) An unpublished vector pBT255.32, containing mut4EGFP [67], [68], was cut by EcoRI and BssSI and yielded a fragment containing a portion of mut4EGFP to reconstitute Syp-mut4EGFP in the final ligation; 3) An unpublished vector pBT248.31 containing the TRE-Bi promoter and tdTomato [17] fused to 3 Myc epitopes (tdT-3Myc) was cut by NheI and EcoRV to place Syp-mut4EGFP under the control of a bidirectional TRE (pBi; Clontech, Mountain View, California; Catalog No. 631006; GenBank Accession No.: U89932). The final ligation product was verified by restriction digestion and sequencing. To generate piiTRE-Bi-SG-Tii, a PmeI/PacI fragment from pTRE-Bi-SG-T was subcloned into pBT226.1. pBT226.1 is a derivative of pJC13-1 [30] and contains a PacI/AscI/PmeI polylinker flanked by two copies of the 1.2 kb-long chicken β-globin HS4 insulator on each side. It was created from pJC13-1 by removal of EcoRI-flanked HS2 enhancer and by replacement of BamHI-flanked Neo reporter by a PacI/AscI/PmeI polylinker. We tested the functionality of pTRE-Bi-SG-T and piiTRE-Bi-SG-Tii constructs by co-transfecting them with a tTA-expressing plasmid into dissociated hippocampal culture (data not shown).

### Transgenesis

**pROSA26-ZtTA** was linearized by AgeI digestion and purified by phenol/chloroform extraction. Gene targeting in mouse R1 ES cells [69] was performed by the Stanford Transgenic Facility. Following G418 selection, clones that contained the knock-in were identified by long-range genomic PCR as previously described [3]. To detect correct targeting at the 5′ end of ROSA26, we amplified ∼1.5 kb genomic DNA fragment using primers: Rosa3 (CCACTGACCGCACGGGGATTC) and Rosa4 (TCAATGGGCGGGGGTCGTT), and LA Taq with GC buffer I (Takara, Cat No. RR02AG). To detect the correct targeting at the 3′ end of ROSA26, we amplified ∼6 kb genomic DNA fragment using primers Rosa8 (GGATCCCCGAATTCTAGATAACTGATCATAATCAGCC) and Rosa9 (GGGGAAAATTTTTAATATAAC), and LA Taq with LA PCR Buffer II (Takara, Cat No. RR002M). Two correctly targeted ES cell clones were expanded and injected into C57BL/6J blastocysts to generate chimeric mice. Germline transmission was verified by PCR as previously described [3]. F1 ZtTA mice were either intercrossed to generate homozygous mice or crossed to Nestin-Cre [39], Nestin-CreER.line1 and Nestin-CreER.line5 [41], or β-actin-CreER [5].

The bacterial plasmid backbone was removed from **pTRE-Bi-SG-T** using PacI and PmeI, and **piiTRE-Bi-SG-Tii** using AccI and NdeI, and separated from the inserts using gel purification. The two constructs were independently introduced into mice to generate randomly integrated transgenes via standard pronuclear injection into FVB zygotes at the Stanford Transgenic Facility. The transgenic mouse founders were identified by PCR genotyping using four primers: (CTTCAAGTCCG CCATGCCCGA) and (TCCAGCAGGACCATGTGATCGC), which detect GFP as a band of 420 bp, and (CAAATGTTGCTTGTCTGGTG) and (GTCAGTCGAGTGCACAGTTT), which detect the control band of 200 bp.

For phenotypic screening, the PCR-positive founders were crossed to mice containing Foxg1-tTA, which is expressed in the mouse forebrain [33].

### Tissue Preparation and Immunohistochemistry

All animal procedures were in compliance with animal care guidelines and were approved by Stanford University's Administrative Panels on Laboratory Care (A-PLAC). Approval number is 14007. For histology, mice were anesthetized with an intraperitoneal injection of 2.5% Avertin (400–600 mg/kg body weight; Sigma, Cat. No. T48402) and perfused intracardially with 0.1 M phosphate buffer saline (PBS; Sigma, Cat. No. P3813) to remove the blood and then with 4% paraformaldehyde (PFA; Sigma, Cat. No. 441244) in 0.1 M PBS. Tissues were isolated and fixed in 4% PFA in 0.1 M PBS at 4°C overnight, washed three times in PBS, cryoprotected for >24 hours in 30% sucrose in PBS, and embedded in Tissue-Tek OCT (VWR, Cat. No 25608-930) prior to cryostat sectioning. Tissues were sectioned at 25 µm or 50 µm thickness unless otherwise specified. 25 µm sections were collected on microscope slides (VWR, Cat. No. 48311-703) and immunostained if needed as described below. 50 µm sections that were to be immunostained were usually collected in PBS and further processed in a “floating” mode. The floating sections were immunostained in 24-well plates following the procedure below and subsequently mounted on slides.

For immunofluorescence, cryosections were washed three times for 10 min in PBS, blocked with 10% normal donkey serum (NDS) in PBS +0.3% Triton X-100 (Sigma, Cat. No. 234729) (PBT) for 1 hour at room temperature, and stained at 4°C overnight with primary antibodies in 5% NDS in PBT. Following four washes for 10 min in PBT, the sections were stained at 4°C overnight with secondary antibodies. The sections were washed four times for 10 minutes with PBT, treated with DAPI (Sigma, Cat. No. D8417), rinsed for 10 minute in PBS, and were mounted in Fluoro-Gel mounting medium (EMS, Hatfield, PA, Cat. No.17985-10). Images were taken using an LSM510 confocal system (Carl Zeiss Inc., Oberkochen, Germany) or a Zeiss fluorescence microscope with a CCD camera and processed using LSM software (Zeiss) and Image J (http://rsbweb.nih.gov/ij/).

Primary antibodies against the following proteins were used: GFP (chicken, 1∶500; Cat. No. GFP-1020; Aves Labs, Tigard, OR), Myc (goat, 1∶200; Cat. No. 600-338; Novus, Littleton, Colorado). Secondary antibodies were obtained from Jackson ImmunoResearch Laboratories, Inc. West Grove, Pennsylvania and included: donkey anti-chicken FITC (1∶200; Cat. No. 703-095-155), donkey anti-goat Cy3 (1∶500, Cat. No. 705-165-147). Pre-absorption of goat anti-Myc antibody (see above) was conducted by cutting three wild-type brains into small pieces after fixation in 4% PFA overnight at 4°C, then blocking the brain pieces at 4°C overnight with 10% NDS in PBT, and finally incubating the fixed brain pieces with shaking in 30 ml of PBT with anti-Myc antibody at 1∶200 dilution for >2 days at 4°C.

### Hippocampal Neuron Culture and FM Dye Staining

Dissociated hippocampal neurons in culture were prepared from P0 mouse pups (genotype: ZtTA/wt; TRE-Bi-SG-T/wt; Nestin-Cre/wt) according to procedures described previously [70] and used between 14 and 43 days *in vitro* (DIV). Briefly, the hippocampal regions were dissected out of brains of triple trangenic mice, cut into small pieces, and incubated in a digestion solution consisting of 10 mg/ml trypsin (Sigma, Cat No. T1005) and 0.5 mg/ml DNAase type IV (Sigma, Cat. No. D5025-15KU). The pieces were then mechanically dissociated in Hank's solution supplemented with 12 mM MgSO_4_ and 0.5 mg/ml DNAase type IV. The cells were recovered by centrifugation and plated onto Matrigel (BD Biosciences -Collaborative biomedical, Cat. No. 35-4234) -coated (6 mg/cm^2^) coverslips (Carolina Biological Supply Company, Cat. No. ER-63-3009) at a density of 25,000–50,000 cells/cm^2^. Culture medium consisted of minimal essential medium (Invitrogen, Cat. No. 51200-038), 0.6% D-glucose, 1 mM NaHCO_3_, 2.4 g/l bovine transferrin (Calbiochem, Cat. No.: 616420), 0.25 g/l insulin (Sigma, Cat No. I6634), 0.3 g/l L-glutamine, 5%–10% fetal calf serum (Invitrogen, Cat. No. 16010-159), and 8 µM Cytosine β-D-arabinofuranoside hydrochloride (Sigma, Cat. No. C6645) Cultures were maintained at 37°C in a 95% air, 5% CO_2_ humidified incubator, and culture medium was replaced every 3 days.

Neurons were loaded with 8 µM FM 4-64 (Invitrogen, Cat. No. F34653) dye solution by field stimulation at room temperature. With field stimulation, neurons were exposed to FM 4-64 during and up to 60 s after stimulation with plantium electrodes (2–120 s, 10 or 20 Hz). After loading, neurons were washed with Tyrode's solution for 5–10 min. Images were taken under a fluorescent microscope (Nikon TE2000) and puncta counting analysis was performed with Metamorph software.

### Array Tomography

One triple transgenic mouse (genotype: ZtTA/wt; BI-TRE-Syp-GFP/wt; Nestin-CreER.line5/wt, with 2 mg tamoxifen injected at E9.5) was perfused at P35 intracardially with 0.1M PBS and 4% PFA in 0.1 M PBS, and the tissue was processed for array tomography [36]. The cerebellum was dissected out, further fixed in the same fixative using microwave irradiation (PELCO 3451 laboratory microwave system; Ted Pella), then dehydrated up to 70% ethanol and embedded in LRWhite resin (medium grade, SPI). Serial ultrathin sections (70 nm) were cut on an ultramicrotome (Leica), mounted on coverslips and immunostained with antibodies against Synapsin I (rabbit, Millipore AB1543P, 1∶100) and MAGUK (mouse, NeuroMabs, 75-029, 1∶100). As secondary antibodies we used Alexa 594 goat anti-mouse, and Alexa 647 goat anti-rabbit (A11032 and A21245, Invitrogen, 1∶150). Sections were mounted using SlowFade Gold antifade reagent with DAPI (Invitrogen). The immunostained serial sections were imaged using a Zeiss AxioImager.Z1 fluorescence microscope with AxioCam HRm CCD camera and a Zeiss 63×/1.4 NA Plan Apochromat objective. Images from serial sections were aligned using ImageJ and the Multistack Reg plugin.

To quantify the colocalization of GFP with endogenous Synapsin and MAGUK, 4 volumes (20×20×2 mm each) from the molecular layer of the cerebellum were analyzed. All GFP puncta that were present on more than one 70 nm section were chosen from a stack of images from the GFP channel. Using the ImageJ ROI function, the same region was then located on the Synapsin channel stack and on the MAGUK channel stack and evaluated for the presence of immunolabeling.

### Quantification of Dox Regulation

Confocal microscopy was used to take a single 8 µm thick optical section of the cerebellum from mice with the genotype of ZtTA/wt; TRE-Bi-SG-T/wt; β-actin-CreER/wt that were raised under different Dox conditions. Three images per animal were taken at random within the granular layer of the cerebellum. Two animals per Dox condition were used for analysis. Average fluorescent intensity within a cell body was measured for every GC body within a given confocal image by using ImageJ after manually positioning the same circle that is smaller than any of the GC bodies within each GC body. For each image, 10 background measurements were applied to normalize the background noise. For each Dox condition, 6 images from 2 animals were used for analysis.

### Quantitative Analysis of Cerebellar Granule Cells

Serial 50 µm coronal sections of cerebellum were immunostained and imaged through a 63× (1.3 NA) oil objective by 0.4 µm optical sectioning using a Zeiss 710 confocal microscope (Carl Zeiss Inc., Oberkochen, Germany). The ascending segments and the parallel fibers were selected that met the following criteria: (1) the entire ascending segment can be followed until the bifurcation point of parallel fiber, (2) the entire cell body and dendritic processes are present, (3) the parallel fibers can be followed within the section (not broken). Given the fact that a single entire parallel fiber (several millimeters long) is always cut into pieces in cryostat sections, it is not practical to trace the entire parallel fiber. Thus for parallel fibers, we traced the axon from the bifurcation points for as long as possible (Figure 4C). Processes (filaments) and synaptic puncta within each section were traced in 3D using the Imaris 6.2 software (Bitplane). Following 3D tracing, the number of synaptic puncta, the filament length between puncta, the total length of the filaments, and the fluorescence intensity of puncta were quantified by using Imaris or ImageJ. Numerical analysis and statistics were performed in Microsoft Excel, Matlab or Graphpad Prism. We calculated the density of synapses (per 100 µm) in a particular region of a GC by taking the total number of puncta in a region/total length of region (in µm) * 100.

For measuring the percentage of Syp-GFP fluorescent signal in a given region of a GC, we processed the confocal stack image of each labeled GC with the “maximal intensity projection” function of ImageJ. Then we measured the total intensity of Syp-GFP for axonal region (X) and the dendritic region (Y), respectively, while excluding the cell body region. We calculated the percentage of Syp-GFP signal for a given GC in the two regions: Syp-GFP_axon_ = X/(X+Y)*100; Syp-GFP_dendrite_ = Y/(X+Y)*100. We applied this type of measurement to both mature and immature GCs (10 cells for each).

## Supporting Information

Figure S1 (A) A summary of expression data for all TRE-Bi-SG-T mouse lines generated in this study. INS−, without insulators; INS+, with insulators. (B) The transgene copy number for the two TRE-Bi-SG-T mouse lines determined by quantitative PCR [71]. GT: an unpublished knock-in mouse line that carries a single copy of GFP and tdT; TT: an unpublished knock-in mouse line that carries two copies of tdT.
(2.81 MB TIF)

Figure S2 Statistics of the distributions of inter-puncta distances in the granule cells axons. (A) A bar graph showing coefficients of variation (CV = SD/mean) of presynaptic densities for all sample groups. Groups are labeled on the X-axis and the CV values on the Y-axis. The density of synapses on the GC ascending segment below and within the PC layer is highly variable between cells (CV between 0.6 and 1.3), while the density of synapses in the GC ascending segments above the PC layer and in parallel fibers is more consistent between cells (CV∼0.2). (B–D) Histograms of the distribution of inter-puncta distances in the ascending segment below and within the PC layer (B), above the PC layer (C) and in parallel fibers (D). The P-value of GOF (Goodness of Fit) is generated by the Chi-Squared test. Two distributions best fit a Burr (4P) distribution (red line) and the other one best fits an Inverse Gaussian (3P) distribution (red line). Both distributions indicate that the inter-puncta distances are assorted randomly. Inter-puncta intervals (in µm) are on the X-axis and the probability density function for observed intervals on the Y-axis.
(0.24 MB TIF)

Figure S3 Statistics of the difference of the mean density for Dox treated and untreated samples. (A) Table listing Dox treatment conditions for all cerebellar granule cell axon segments analyzed in Figure 4E and 4F. Cells are listed in the order they appear from top to bottom in the plots in Figure 4. (B) Quantification of Syp-GFP mean density (the number of puncta per 100 µm) for Dox treated and untreated samples. Each green dot represents a data point. A black line marks the mean of each column. Error bars are ± standard error of the mean (SEM). There is no significant difference between Dox treated and untreated samples in a given region of the GC (Mann Whitney; below PC, p = .3726; PC, p = .4121; above PC, p = .7921; PF, p = .3311), suggesting that Syp-GFP expression in untreated samples does not significantly alter synaptic density. (C) Histogram of the coefficient of variation (CV = SD/mean) of presynaptic densities for Dox treated and untreated samples in all regions of the GC. Groups are labeled on the X-axis and CV values on the Y-axis. The CV varies little between Dox treated and untreated samples, indicating that Syp-GFP expression in the untreated condition does not affect presynaptic density.
(6.74 MB TIF)
