# Supplementary figures and images for: Visualizing the Distribution of Synapses from Individual Neurons in the Mouse Brain
Source: PLoS One. 2010 Jul 9;5(7):e11503. doi: 10.1371/journal.pone.0011503 (PMC2901335; doi:10.1371/journal.pone.0011503)

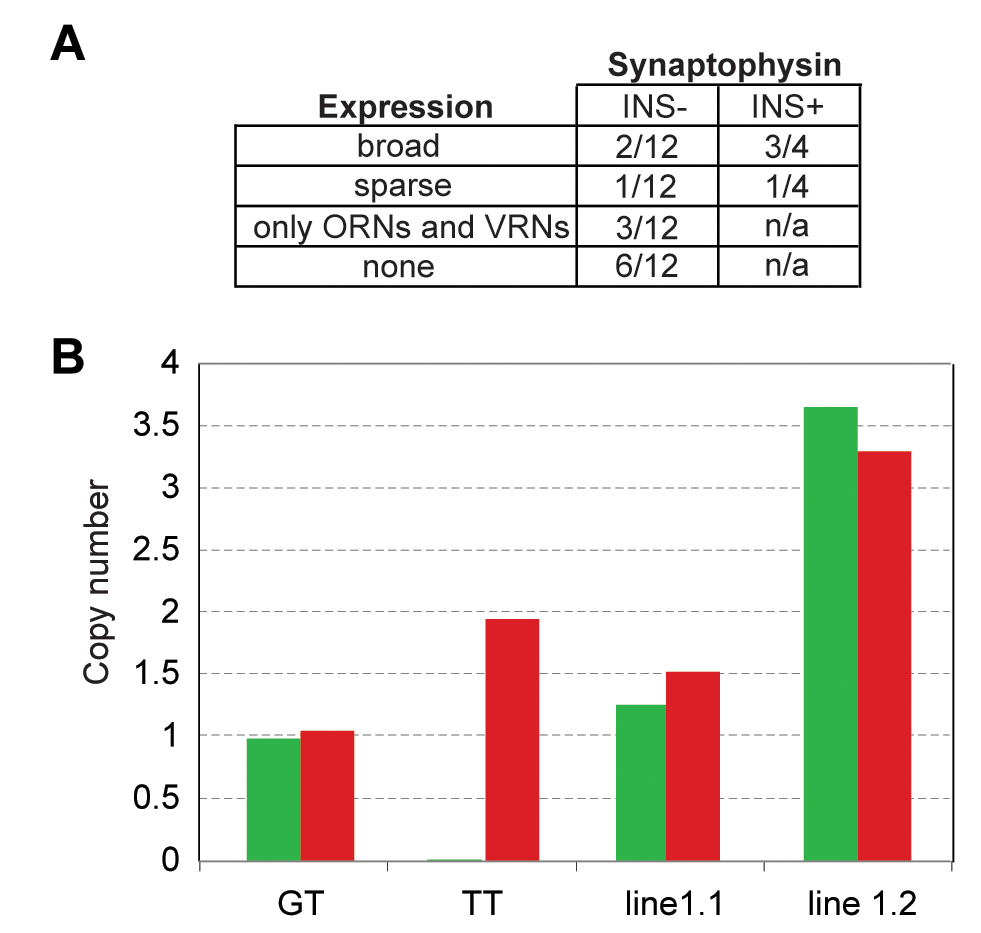

Supplement: Figure S1 — (A) A summary of expression data for all TRE-Bi-SG-T mouse lines generated in this study. INS−, without insulators; INS+, with insulators. (B) The transgene copy number for the two TRE-Bi-SG-T mouse lines determined by quantitative PCR [71]. GT: an unpublished knock-in mouse line that carries a single copy of GFP and tdT; TT: an unpublished knock-in mouse line that carries two copies of tdT. (2.81 MB TIF) [file pone.0011503.s001.tif]

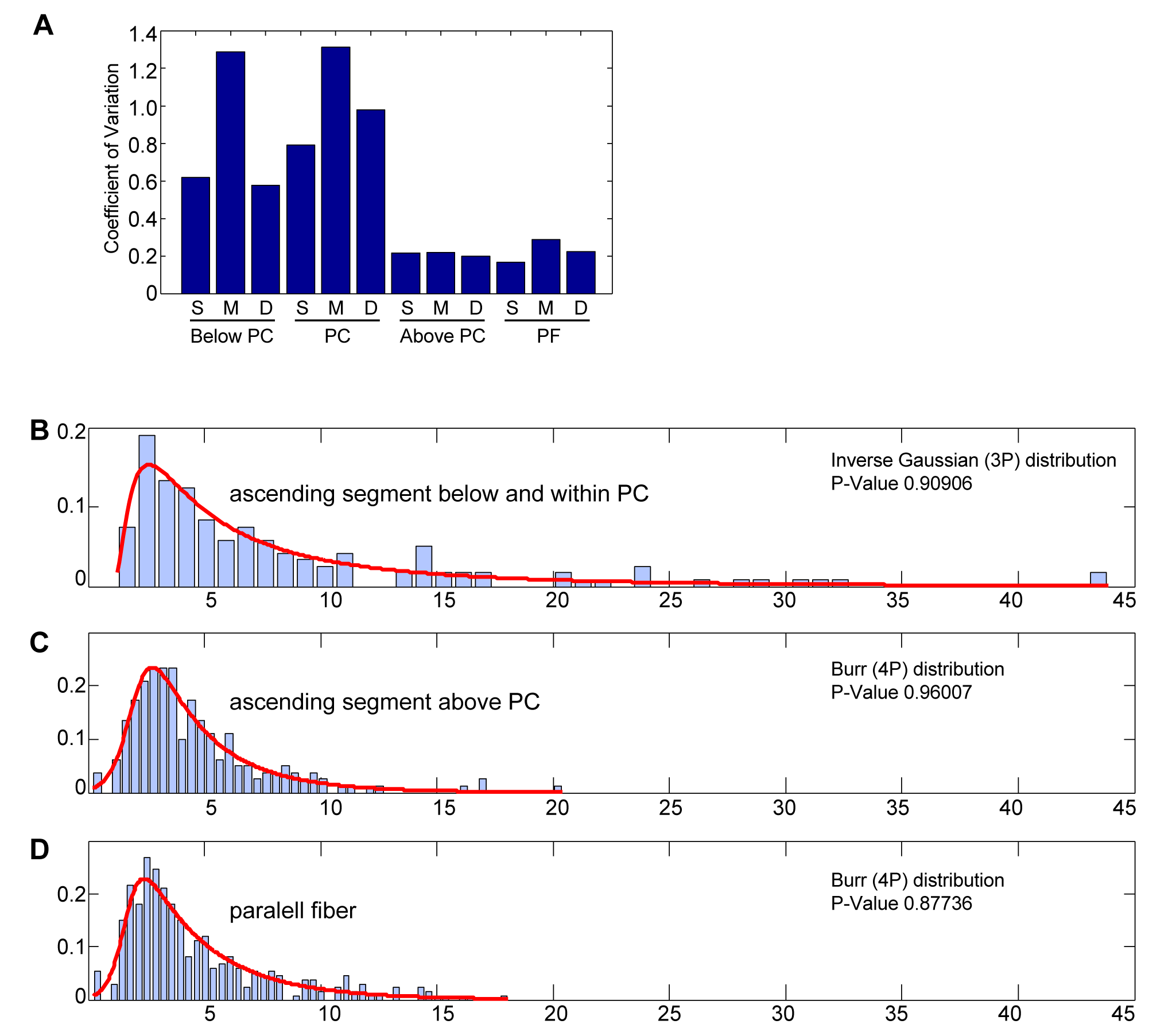

Supplement: Figure S2 — Statistics of the distributions of inter-puncta distances in the granule cells axons. (A) A bar graph showing coefficients of variation (CV = SD/mean) of presynaptic densities for all sample groups. Groups are labeled on the X-axis and the CV values on the Y-axis. The density of synapses on the GC ascending segment below and within the PC layer is highly variable between cells (CV between 0.6 and 1.3), while the density of synapses in the GC ascending segments above the PC layer and in parallel fibers is more consistent between cells (CV∼0.2). (B–D) Histograms of the distribution of inter-puncta distances in the ascending segment below and within the PC layer (B), above the PC layer (C) and in parallel fibers (D). The P-value of GOF (Goodness of Fit) is generated by the Chi-Squared test. Two distributions best fit a Burr (4P) distribution (red line) and the other one best fits an Inverse Gaussian (3P) distribution (red line). Both distributions indicate that the inter-puncta distances are assorted randomly. Inter-puncta intervals (in µm) are on the X-axis and the probability density function for observed intervals on the Y-axis. (0.24 MB TIF) [file pone.0011503.s002.tif]

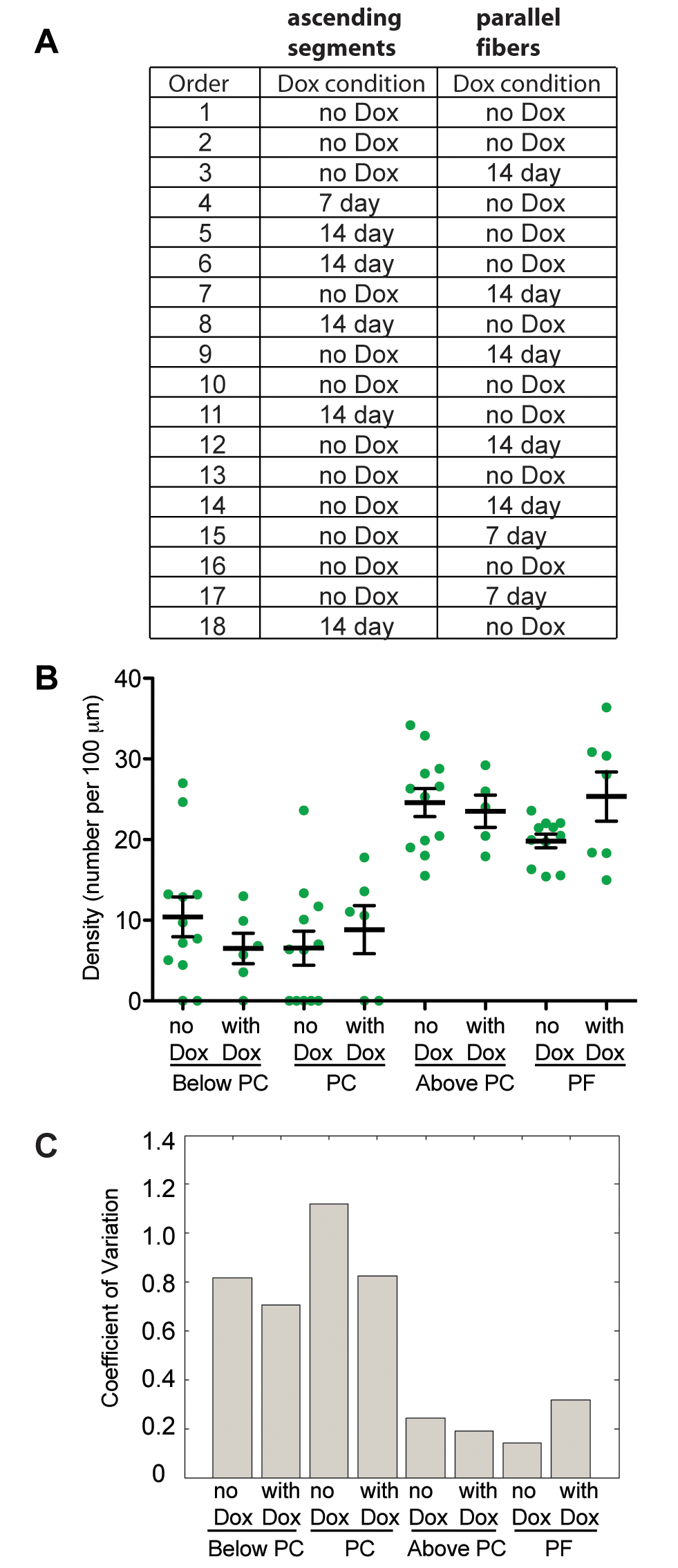

Supplement: Figure S3 — Statistics of the difference of the mean density for Dox treated and untreated samples. (A) Table listing Dox treatment conditions for all cerebellar granule cell axon segments analyzed in Figure 4E and 4F. Cells are listed in the order they appear from top to bottom in the plots in Figure 4. (B) Quantification of Syp-GFP mean density (the number of puncta per 100 µm) for Dox treated and untreated samples. Each green dot represents a data point. A black line marks the mean of each column. Error bars are ± standard error of the mean (SEM). There is no significant difference between Dox treated and untreated samples in a given region of the GC (Mann Whitney; below PC, p = .3726; PC, p = .4121; above PC, p = .7921; PF, p = .3311), suggesting that Syp-GFP expression in untreated samples does not significantly alter synaptic density. (C) Histogram of the coefficient of variation (CV = SD/mean) of presynaptic densities for Dox treated and untreated samples in all regions of the GC. Groups are labeled on the X-axis and CV values on the Y-axis. The CV varies little between Dox treated and untreated samples, indicating that Syp-GFP expression in the untreated condition does not affect presynaptic density. (6.74 MB TIF) [file pone.0011503.s003.tif]
